# Excessive BMI is associated with higher C-peptide level at recognition but also with its greater loss in two years clinical observation in children with new onset type 1 diabetes

**DOI:** 10.3389/fimmu.2023.1176403

**Published:** 2023-04-27

**Authors:** Emilia Kurpiewska, Sebastian Ciężki, Milena Jamiołkowska-Sztabkowska, Agnieszka Polkowska, Aleksandra Starosz, Kamil Grubczak, Marcin Moniuszko, Artur Bossowski, Barbara Głowińska-Olszewska

**Affiliations:** ^1^ Department of Pediatrics, Endocrinology, and Diabetology with Cardiology Division, Medical University of Bialystok, Białystok, Poland; ^2^ Department of Regenerative Medicine and Immune Regulation, Medical University of Bialystok, Białystok, Poland; ^3^ Department of Allergology and Internal Medicine, Medical University of Bialystok, Białystok, Poland

**Keywords:** diabetes type 1, obesity, children, cytokines, C-peptide, residual beta cell function

## Abstract

**Introduction:**

The prevalence of obesity in general pediatric population increases without sparing children with T1D. We intended to find factors associated with the possibility of preserving endogenous insulin secretion in individuals with long-standing T1D. At onset, higher BMI is associated with higher C-peptide level, which may indicate to be one of the favorable factors involved in preserving residual β-cell function. The study determines the influence of BMI on C-peptide secretion in children newly diagnosed with T1D in two years observation.

**Methods:**

We assessed the possible relationship between selected pro- and anti-inflammatory cytokines, body mass at recognition and β-cell function status. 153 pediatric patients with newly diagnosed T1D were divided into quartiles according to BMI-SDS index. We separated a group consisted of patients with BMI-SDS >1. Participants were followed up for two years and examined for changes in body weight, HbA1c, and insulin requirement. C-peptide was assessed at baseline and after two years. We evaluated the patients’ levels of selected inflammatory cytokines at baseline.

**Results:**

Subjects with higher BMI-SDS presented higher serum C-peptide levels and lower insulin requirements at diagnosis than children with lower body weight. The two-year follow-up showed that C-peptide levels of obese patients dropped more rapidly than in children with BMI-SDS within normal limits. The group with BMI-SDS >1 showed the greatest decrease in C-peptide level. Despite statistically insignificant differences in HbA1c at diagnosis between the study groups, in the fourth quartile and BMI-SDS >1 groups, HbA1c as well as insulin requirements increased after two years. The levels of cytokines varied the most between BMI-SDS <1 and BMI-SDS >1 groups and were significantly higher within BMI-SDS >1 group.

**Discussion:**

Higher BMI, associated with enhanced levels of inflammatory cytokines, relates to preservation of C-peptide at T1D recognition in children but is not beneficial in the long term. A decrease in C-peptide levels combined with an increase in insulin requirements and in HbA1c among patients with high BMI occur, which may indicate a negative effect of excessive body weight on the long term preservation of residual β-cell function. The process seems to be mediated by inflammatory cytokines.

## 1 Introduction

Within the recent decades the incidence of both excessive body mass (1) and diabetes (2) became an increasing problem in the pediatric population. Along with the growing prevalence of overweight and obesity in children suffering from type 1 diabetes (T1D) (3–5), the interest in the coexistence and mutual correlation between these disease entities increases. As a result, a new term- double diabetes, has emerged to characterize patients with T1D who also exhibit clinical features of type 2 diabetes (T2D), such as insulin resistance or obesity (6).

In general, T1D is described as a progressive autoimmune destruction of pancreatic β-cells, leading to an absolute insulin insufficiency (7). However, numerous studies have shown that a great number of patients with long-duration T1D endure to secrete low levels of endogenous insulin (8, 9). This may imply persistence of a small number of still functioning β-cells. The measurement of serum C-peptide levels (which is produced in equal amounts to insulin) remains the best method to estimate residual β-cells insulin secretion (8, 10). Studies have demonstrated that even low persistent C-peptide secretion exhibit a positive impact on metabolic control (lower HbA1c concentrations, glucose variability, incidence of hypoglycaemia and insulin demand) and a protective effect on acute and long-term diabetes complications (11, 12).

Moreover, C-peptide levels presented in some studies as an independent factor of partial remission and is correlated with its duration (13, 14). The phenomenon of partial remission (PR), also known as the honeymoon phase, occurs in a number of patients who experience a transient reduction in insulin requirements in parallel with achieving proper metabolic control after the initiation of effective insulin therapy (15). PR is defined as a daily insulin requirement (DIR) of <0.5 units per kg of body weight and HbA1c <7% (16). Although the mechanisms responsible for the occurrence of this phenomenon are not yet well investigated, it appears that partial β-cell recovery with improved endogenous insulin production, transient recovery of immune tolerance and reduced peripheral insulin resistance may play an underlying role (17). In recent decades, attempts have been made to preserve β-cell function in patients with type 1 diabetes because of the clear clinical benefits of the PR period (17). Therefore, novel therapies have emerged to prolong the period of PR, such as cell therapies using the patient’s own proliferated regulatory T cells (Tregs) or umbilical cord stem cells, immunomodulatory therapies or granulocyte-colony stimulating factor (G-CSF), but unfortunately, only some of them can be implemented in pediatric patients (10). It has been observed that younger age and higher BMI at recognition are factors that potentially predispose to PR in children suffering from type 1 diabetes (18). In turn, in our previous study we found that regular physical activity promotes prolonged PR time in the pediatric diabetes (17).

Nevertheless, C-peptide levels are firmly and independently related to body weight and age at and shortly after the diagnosis of T1D (19). There are several studies concerning the β-cell secretory function in obese T1D individuals revealing that children with obesity have higher C-peptide levels than their overweight and lean peers (20–22). Noteworthy is also the fact that even with adjusting for glycemia, higher BMI at diagnosis was associated with greater fasting C-peptide levels decrease, thus more rapid disease progression among pooled European cohorts of teens with T1D over one year of follow-up (23). To the best of our knowledge, this is the one and only observation regarding the effect of BMI on the rate of β-cell loss, with an observation period lasting only one year. Interestingly, study of a group of young children diagnosed with type 1 diabetes found that greater C-peptide decline during the first two years after diagnosis was associated with female gender, younger age at diagnosis, higher weight Z-score, and higher HbA1c, as well as positive IA-2 and ZnT8 antibodies (24). Moreover, if the centrally and noncentrally obese groups of children are compared, the centrally obese youths have remarkably higher secretion of C-peptide at onset but not at follow-up (21).

Intriguingly, some studies have shown that higher BMI is related to elevated levels of pro-inflammatory cytokines, which could potentially affect the destruction of pancreatic islets (25–29). The fact that β-cell destruction occurring in the course of obesity is not only a consequence of insulin resistance but is also triggered by the devastating effects of pro-inflammatory cytokines sheds new light on understanding the causes of the negative effect of obesity on residual β-cell function in type 1 diabetes. Taking together, these data suggest that BMI-associated insulin resistance and inflammation contributes to β-cell exhaustion and that healthy adipose tissue is crucial for β-cell function.

In our study we hypothesized that greater BMI is associated with higher C-peptide level at diagnosis, but during follow-up C-peptide levels dramatically decrease along with reduced residual pancreatic β-cell function. However, few studies evaluating the effects of obesity-associated inflammatory cytokines on residual β-cell function have been done so far, thus we decided to investigate this relationship.

## 2 Material and methods

Our prospective observational research covered 153 youths newly diagnosed with T1D in the Department of Pediatrics, Endocrinology, and Diabetology with Cardiology Division, Medical University of Bialystok, Poland, who were consistently examined trimonthly in Outpatient Clinic during two years. Every patient received insulin intravenously at the time of diagnosis. The therapy has been changed to functional intensive therapy (multiple daily injections (MDIs) or continuous subcutaneous insulin infusion (CSII)) as the method of choice when the patient’s condition was stabilized. The main inclusion criteria were ISPAD diagnosis criteria of type 1 diabetes and age under 18 years. Exclusion criteria were diagnosis of other types of diabetes, such as MODY and type 2 diabetes. The patients were encouraged to maintain their normal physical activity in accordance with ISPAD recommendations (30).

Anthropometric data such as gender, height, weight and body mass index (BMI) have been assessed. To adjust for age and gender, the BMI SD score (BMI-SDS) was calculated using sex-specific and age-specific BMI growth charts based on local Polish OLAF study (31). All the subjects were positive for at least one of the anti-GAD, IA2, ICA or ZnT8 antibodies. Regarding BMI-SDS, participants were divided into four groups (quartiles of BMI-SDS within the study group). We also separated a group consisted of patients with BMI-SDS >1 (overweight and obese according to centile population charts) (n=22). All participants were followed up trimonthly for the next two years and assessed by examination for changes in body weight, HbA1c [%] and daily insulin requirement (DIR) [U/kg/24h].

DIR mean values were calculated depending on the information collected during the medical appointments from the course of previous 2 weeks, from patient’s home monitoring diary or personal insulin pump reports. Total DIR and HbA1c at the disease onset, as well as one year and two years after diagnosis were evaluated. The analyzed results of fasting C-peptide levels on the first admission to hospital and after two-years of observation are given as [ng/mL] and a cut-off level of clinical validity was adjusted from 0.2 nmol/L to 0.6 ng/mL (32).

Levels of cytokines were determined once, at disease onset as previously described and published (33). Plasma samples collected from patients were used to analyze concentrations of the selected cytokines. Immunoenzymatic assays were performed in accordance with the manufacturer protocols (DuoSet ELISA Kits; R&D system). The following cytokines were analyzed in patients’ and healthy controls’ plasma: IL-1β, IL-10, IL-12p70, IL-17, IFN-γ, TNF-α. Cytokines concentration-related absorbance was measured at wavelength of 450nm using LEDETECT96 microplate reader (Labexim Products, Lengau, Austria). Standard curve was performed based on the four-parameter logistic (4-PL) curve-fit, and subsequently used to estimate the final cytokines’ concentration.

After the patients had recovered from ketoacidosis, CRP and morphology tests were performed as part of standard clinical procedure along with other laboratory tests. The results were within the normal range and precluded a clinical diagnosis of inflammation at that point.

## 3 Statistical analysis

Statistical analysis was performed using Statistica version 13.3 (StatSoft Krakow, Poland). The Kolmogorov-Smirnov test of normality was used to test the distribution of all continuous variables with Lillefors correction and Shapiro-Wilk tests. All examined variables had a parametric distribution and were evaluated using the unpaired Student t-test to compare the differences between the groups and as several of the studied parameters were not normally distributed, the two-tailed Mann-Whitney U-test was used to correlate continuous variables. In the analysis of more than two groups, the analysis of variance was used with Tukey’s *post-hoc* RIR test for unequal numbers or the ANOVA rang Kruskal-Wallis test and the median test with *post-hoc* tests of multiple comparisons. All data are expressed as mean ± SD. Relative risk was evaluated with the use of Fishers’ exact test, and calculation of 95% confidence interval with Koopman asymptomatic score. We performed Pearson correlation analysis to analyse possible associations of intensity of pancreatic autoimmunity (GADA, ICA, IA2) and BMI, BMI-SDS, HbA1c, DIR and C-peptide. We also performed an analysis of covariance (ANCOVA) including gender and age as the accompanying variables to verify their possible influence on the ANOVA results between groups stratified by BMI-SDS into quartiles.

Differences were considered statistically significant with p-value below 0.05. Significant changes were demonstrated within the graphs or tables with asterisks or exact p-value: * - p < 0.05, ** - p < 0.01, *** - p < 0.001, **** - p < 0.0001.

## 4 Ethical approval

We obtained approval of the Ethical Committee at the Medical University of Bialystok (R-I-002/435/2017). Written informed consent was obtained from parents/legal guardians as well as from patients aged > 16 years.

## 5 Results

The study included 153 Caucasian children with a proportional ratio of both sexes (49% boys). The clinical and biochemical characteristics of the study group are collected in **Table 1**.

**Table 1:** The clinical and biochemical characteristics of all patients and regarding to groups according to BMI-SDS.

	All patients	BMI-SDS Q_1_	BMI-SDS Q_2_	BMI-SDS Q_3_	BMI-SDS Q_4_	BMI-SDS >1
**No. of patients**	153	39	38	38	38	22
**Male, %**	49%	46%	52%	50%	52%	54%
**Age at onset [years]**	8.67 ± 4.54	8.64 ± 5.22	7.93 ± 4.54	9.40 ± 3.72	8.70 ± 4.60	9.00 ± 4.23
**pH value at onset**	7.33 ± 0.10	7.30 ± 0.11	7.35 ± 0.09	7.35 ± 0.10	7.31 ± 0.11	7.30 ± 0.10
**BMI-SDS at onset**	-0.29+1.20	-1.68 ± 0.51	-0.72 ± 0.29	-0.01 ± 0.25	1.31 ± 0.75	1.79 ± 0.63
**HbA1c at onset [%]**	11.47 ± 2.65	11.92 ± 2.79	11.40 ± 2.59	11.40 ± 2.57	11.13 ± 2.69	11.35 ± 2.66
**DIR at discharge [U/kg/24 h]^a,b,c,f^**	0.57 ± 0.24	0.69 ± 0.26	0.55 ± 0.24	0.53 ± 0.22	0.50 ± 0.21	0.47 ± 0.19
**Fasting C-peptide at onset [ng/mL] ^b,c,d,e,f,g^**	0.55 ± 0.40	0.44 ± 0.23	0.45 ± 0.30	0.62 ± 0.40	0.73 ± 0.54	0.77 ± 0.65
**HbA1c after 1 year [%]**	6.61 ± 0.88	6.55 ± 0.67	6.54 ± 0.84	6.57± ± 1.04	6.78 ± 0.98	6.53 ± 0.91
**DIR after 1 year [U/kg/24 h]**	0.59 ± 0.21	0.61 ± 0.17	0.59 ± 0.25	0.60 ± 0.22	0.56 ± 0.22	0.55 ± 0.26
**HbA1c after 2 years [%]^a,b,c,f^**	6.70 ± 1.00	6.39 ± 0.70	6.81 ± 0.96	6.62 ± 1.05	6.99 ± 1.22	7.02 ± 1.14
**DIR after 2 years [U/kg/24 h]**	0.70 ± 0.23	0.70 ± 0.26	0.72 ± 0.24	0.66 ± 0.21	0.71 ± 0.24	0.75 ± 0.27
**Fasting C-peptide after 2 years [ng/mL] ^b^**	0.46 ± 0.46	0.37 ± 0.37	0.38 ± 0.31	0.58 ± 0.57	0.46 ± 0.52	0.58 ± 0.76
**% decrease in c-peptide level during the time of observation**	16.36%	15.91%	15.56%	6.45%	36.99%	24.68%
**IL-1beta/IL-1F2 [pg/mL]^f^**	145.87 ± 184.80	79.77 ± 101.46	173.10 ± 241.48	129.47 ± 158.54	196.47 ± 198.75	268.37 ± 234.41
**IL-10 [pg/ml] ^f^**	824.44 ± 1187.98	511.52 ± 616.93	991.48 ± 1624.49	586.62 ± 655.86	1140.21 ± 1390.27	1646.24 ± 1673.64
**IL-12p70 [pg/ml] ^f,h^**	667.09 ± 1066.32	297.70 ± 326.29	981.01 ± 1593.33	390.63 ± 470.80	920.03 ± 1131.94	1482.59 ± 1378.68
**IL-17 [pg/ml] ^f^**	298.56 ± 424.63	163.61 ± 228.36	343.28 ± 549.79	244.56 ± 226.34	427.37 ± 525.00	595.47 ± 613.69
**TNF-alpha [pg/ml] ^f^**	270.55 ± 530.79	103.84 ± 113.12	425.19 ± 851.85	171.29 ± 277.61	353.51 ± 492.76	537.13 ± 596.38
**IFN-gamma [pg/ml] ^f^**	123.37 ± 84.98	104.64 ± 78.45	129.67 ± 101.77	114.66 ± 98.66	142.05 ± 65.03	182.35 ± 54.75

Results are presented as number, percent or mean values ± standard deviation. Superscript letters beside parameters means P values <.05, where:

^a^statistically significant difference between 1^st^ and 2^nd^ quartile.

^b^statistically significant difference between 1^st^ and 3^rd^ quartile.

^c^statistically significant difference between 1^st^ and 4^th^ quartile.

^d^statistically significant difference between 2^nd^ and 3^rd^ quartile.

^e^statistically significant difference between 2^nd^ and 4^th^ quartile.

^f^statistically significant difference between 1^st^ quartile and BMI-SDS >1 group.

^g^statistically significant difference between 2^nd^ quartile and BMI-SDS >1 group.

^h^statistically significant difference between 3^rd^ quartile and BMI-SDS >1 group.

BMI, body mass index; BMI-SDS, BMI SD score; DIR, daily insulin requirement; Q, quartile.

**Figure 1:**
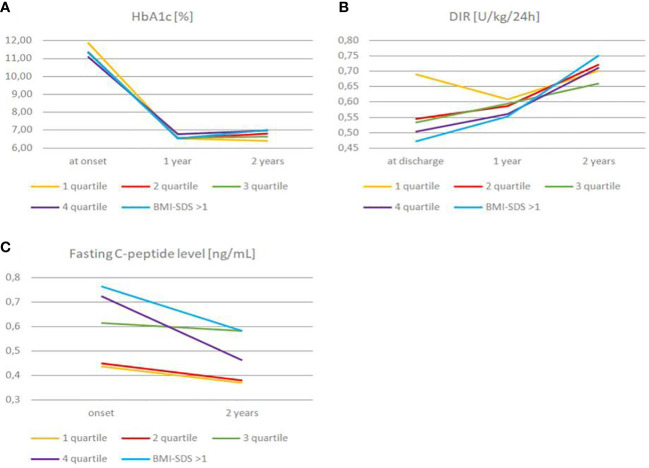
Changes in HbA1c **(A)**, daily insulin requirement **(B)** and fasting C-peptide level **(C)** among quartiles and BMI-SDS >1 group over the observation time.

First, we analyzed differences of HbA1c levels between the groups at the time of diagnosis, after a year and two years (**Figure 1A**). *We found that average* HbA1c at the first admission to the hospital among all the groups was comparable, with mean value 11.46 ± 2.65%. Nevertheless, after two years there were noticeable differences in HbA1c levels, especially in comparison of first and fourth BMI-SDS quartile groups (6.39% vs. 6.99%, P = .014). We have also observed a slight difference in HbA1c in BMI-SDS<1 an BMI-SDS>1 groups (6.64% vs. 7.02%, P = .14) (**Tables 1** and **2**).

**Table 2:** The comparison of the mean values of parameters affecting c- peptide level between the analyzed groups of patients divided according to SDS-BMI below and over 1 at the disease onset, after one year and two years of observation.

Parameter	SDS-BMI <1	SDS-BMI >1	P value
**No. of patients**	131	22	–
**Male, %**	50%	54%	–
**Age at onset [years]**	8.62	9.00	0.714
**pH value at onset**	7.33	7.30	0.229
**BMI-SDS at onset**	**-0.63**	**1.79**	**0.000**
**HbA1c at onset [%]**	11.49	11.35	0.823
**DIR at discharge [U/kg/24 h]**	**0.58**	**0.47**	**0.044**
**Fasting C-peptide at onset [ng/mL]**	**0.52**	**0.77**	**0.009**
**HbA1c after 1 year [%]**	6.62	6.53	0.673
**DIR after 1 year [U/kg/24 h]**	0.59	0.55	0.501
**HbA1c after 2 years [%]**	6.65	7.02	0.139
**DIR after 2 years [U/kg/24 h]**	0.69	0.75	0.314
**Fasting C-peptide after 2 years [ng/mL]**	0.44	0.58	0.291
**% decrease in c-peptide level during the time of observation**	15.39%	24.68%	–
**BMI-SDS after 2 years**	**0.37**	**1.79**	**0.000**
**IL-1beta/IL-1F2 [pg/mL]**	126.82	268.37	0.059
**IL-10 [pg/ml]**	**696.61**	**1646.24**	**0.048**
**IL-12p70 [pg/ml]**	**540.23**	**1482.59**	**0.028**
**IL-17 [pg/ml]**	**252.37**	**595.47**	**0.046**
**TNF-alpha [pg/ml]**	229.08	537.13	0.155
**IFN-gamma [pg/ml]**	**114.20**	**182.35**	**0.047**

Results are presented as mean values. P values <.05 are in bold.

BMI, body mass index; BMI-SDS, BMI SD score; DIR, daily insulin requirement.

DIR of the study groups at hospital discharge and two years after diagnosis was also compared (**Figure 1B**). The mean DIR at the time of diagnosis in the whole study group was 0.57U/kg/24h. Initial DIR differed significantly between the first and fourth quartile (0.69 vs. 0.50 U/kg/24h, P = .01). Interestingly, we found no significant differences in DIR between the first and fourth quartile two years after diagnosis (0.70 vs. 0.71 U/kg/24h, P = .877). Similarly, a substantial difference in DIR at the disease onset was noted when comparing the group with BMI-SDS<1 and BMI-SDS>1 (0.58 vs. 0.47 U/kg/24h P = .0.044), while two years after diagnosis the difference was statistically insignificant (P = .31) (**Table 2**).

We also observed that during one year of follow-up, between the first and second year of observation, HbA1c and DIR increased in the subgroup of children within BMI-SDS>1. HbA1c level increased from 6.53% to 7.02% (P = .152) and DIR from 0.55 to 0.75 U/kg/24h (P = .045). It should be added that this group of children maintained their weight throughout the observation period (average BMI-SDS value sustained 1.79).

Next, we analyzed fasting C-peptide secretion in our patients at admission and two years after diagnosis (**Figure 1C**). The mean fasting C-peptide level (presented results are corrected for age and gender) on admission in the entire study group was 0.55 (0.44-0.73) ng/mL. There was a meaningful difference between the fasting C-peptide level among first and fourth quartiles on admission (0.44 vs. 0.73 ng/mL, P = .002), while two years after diagnosis this difference has become marginal (P = .39). We obtained similar results when comparing groups of patients with BMI-SDS >1 and BMI-SDS<1. At the time of admission, there was a substantial difference in fasting C-peptide levels between the BMI-SDS<1 and BMI-SDS >1 groups (0.52 vs. 0.77 ng/mL, P = .009), respectively two years after, we observed only a slight difference (P =.29) (**Table 2**). We noted a 36.99% decrease in C-peptide level in the fourth quartile group and 24.68% decrease in BMI-SDS >1 group over two years of follow-up. Interestingly, the lowest decrease in the level of C-peptide (only by 6.45%) was noted in the third quartile (BMI-SDS -0.01 ± 0.25) (**Table 1**).

**Figure 2:**
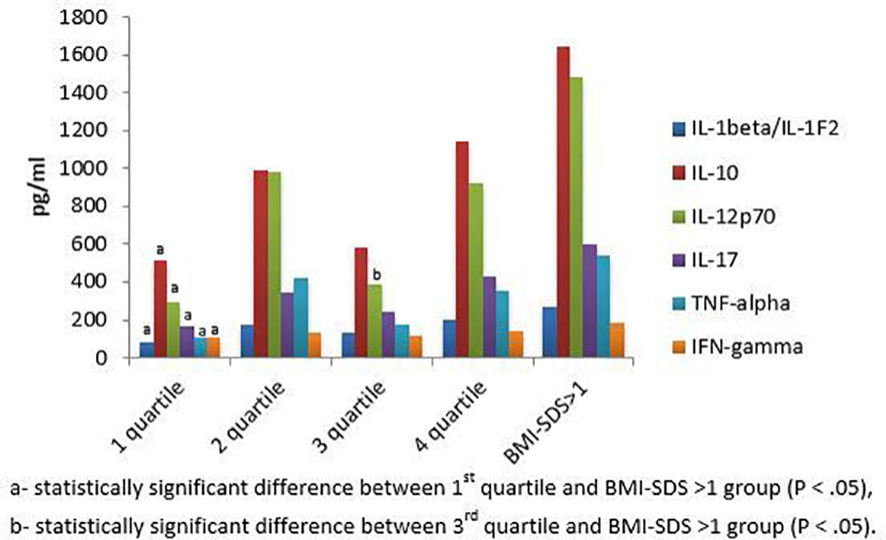
Mean values of inflammatory cytokines levels at T1D onset among quartile and BMI-SDS >1 groups.

Eventually, we measured patients’ levels of inflammatory cytokines such as IL-1β, IL-10, IL-12p70, IL-17, TNF-alpha and IFN-gamma at the time of diagnosis (**Figure 2**). We observed a statistically significant difference in the level of IL-10 (696.6 vs. 1646.2 pg/ml, P = .048), IL-12p70 (540.23 vs. 1482.6 pg/ml, P = .028), IL-17 (252.4 vs. 595.5, P = .046), and IFN-gamma (114.20 vs. 182.35 pg/ml, P= .047) between BMI-SDS <1 and BMI-SDS >1 groups, respectively (**Table 2**). However, differences between these groups in the level of IL-1β and TNF-alpha were marginal (P = .059, P = .155, respectively). Within 1st, 2nd and 4th quartile, mean levels of IL-1β, IL-10, IL-17 and IFN-gamma elevate with increasing BMI-SDS, although with no statistical significance (P >.05) (**Table 1**; **Figure 2**). Interestingly, children among 3rd quartile showed lower levels of all cytokines in comparison to those within 2nd quartile as well as their greater amounts when considering patients in 1st quartile. We correlated the levels of inflammatory cytokines with C-peptide levels among the subjects; however, we did not obtain a statistically significant resulst. Nevertheless, this relationship, in our opinion, requires further research.

Our analyses show that groups with BMI-SDS>1 and BMI-SDS<1 did not statistically differ in age (P = 0.71), pH (P = 0.23) and base excess (P = 0.1) at the time of diagnosis. Moreover, we observed that differences between age and pH at admission between the first and fourth quartiles were not substantial (P = 0.96 and P = 0.75, respectively). All the mean values of the BMI-SDS <1 and >1 groups characteristics including parameters affecting C-peptide level as well as their comparison with respect to the P value between groups at the disease onset, after one year and two years of observation are presented within **Table 2**.

Correlation analysis adjusted for age considering the possible associations of intensity of pancreatic autoimmunity (GADA, ICA, IA2) with BMI-SDS, BMI, HbA1c and C-peptide showed no statistical significance between studied autoimmunity and other variables within the entire study group. However, in correlation analysis adjusted for age performed between BMI-SDS at onset and inflammatory cytokines we found significant correlation with IL-10 (r = .316 P = .033) as well as with IL-17 (r = -.312; P = .035). Additionally, BMI-SDS correlated significantly with C-peptide at onset (r = .367; P <0.001), DIR at discharge (r = -.270; P = .001) and HbA1c after 2 years (r = .179; P = .031) (**Table 3**).

**Table 3:** Correlation analysis between BMI-SDS at onset and studied variables adjusted for age within the entire study group.

Parameter	BMI-SDS at onset
**Age at onset**	r=0.074; P=0.363
**pH value at onset**	r=0.058 P=0.474
**HbA1c at onset**	r=-0.093; P=0.252
**DIR at discharge**	**r=-0.270; P=0.001**
**Fasting C-peptide at onset**	**r=0.367; P<0.001**
**HbA1c after 2 years**	**r=0.179; P=0.031**
**DIR after 2 years**	r=0.001; P=0.988
**Fasting C-peptide after 2 years**	r=0.146; P=0.155
**IL-1beta/IL-1F2**	r=0.208; P=0.166
**IL-10**	**r=0.316 P=0.033**
**IL-12p70**	r=-0.242; P=0.105
**IL-17**	**r=-0.312; P=0.035**
**TNF-alpha**	r=0.239; P=0.109
**IFN-gamma**	r=0.187; P=0.214
**GADA**	r=0.084; P=0.313
**ICA**	r=-0.091; P=0.274
**IA2A**	r=0.052; P=0.531

P values <.05 are in bold.

Interestingly, there are some statistically significant correlations between GADA and BMI-SDS at onset within particular sub-groups: in the BMI-SDS 4^th^ quartile group (r = .378; P = .021), in the group above the median BMI-SDS (r = .283; P = .017), and similar trend for the BMI-SDS>1 (r = .457; P = .037).

**Figure 3:**
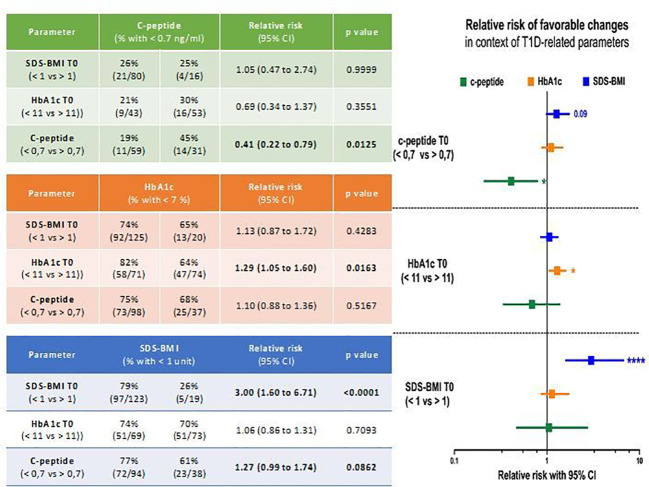
Risk assessment of selected parameters’ values prevalence at 2^nd^ year of diabetes therapy using laboratory data obtained at patients’ admission stage. Achievement of clinically favorable changes was based on normal ranges of selected parameters: < 1 for SDS-BMI, < 7% for HbA1c, > 7ng/ml for c-peptide. Significant results indicated with asterisks or exact p value: * - p < 0.05, ** - p < 0.01, *** - p <0.001, **** - p < 0.

In our final analysis, we intended to evaluate predictive potential of selected diabetes-related parameters in establishing outcome of the therapy. First, we observed that patients with values of c-peptide lower than 0.7 ng/ml at the admission stage had significantly higher risk of keeping that parameter below clinically significant level after 2-year treatment (p = 0.0125). On the other hand, the same group of pediatric subjects had slightly higher tendency for maintaining normal level of SDS-BMI (p = 0.0862). As presumed, those patients who demonstrated initially SDS-BMI values of above 1, had substantially higher risk of that parameter elevated also after 2 years of therapy (p < 0.0001). Similarly, for subjects with lower median HbA1c levels (below 11%) at admission, the odds for these patients for reaching HbA1c normalization were significantly increased (p = 0.0163) (**Figure 3**).

## 6 Discussion

The current study was designed to determine the impact of BMI on the preservation of residual β-cell function in children with newly diagnosed type 1 diabetes. We found a positive correlation between BMI-SDS and serum C-peptide level in our patients, occurring only at the time of diagnosis. Interestingly, after two years of follow-up, we observed a sharp decrease in serum C-peptide level, as well as an increase in daily insulin requirement and HbA1c in the group of children with the highest BMI-SDS, indicating that in long-term observation higher BMI affects residual β-cell function negatively. Previous studies support our finding that at the time of diagnosis, children with higher BMI-SDS demonstrate more preserved β-cell function than thinner ones. Szypowska et al. in their study revealed that obese and overweight children show higher fasting C-peptide levels as well as lower HbA1c levels compared to leaner individuals at the baseline (34). In addition, Redondo et al. demonstrated that the likelihood of having a preserved C-peptide was 2.4 fold and 4.1 fold higher in overweight and obese children, respectively, compared to their lean peers at the disease onset (35).

In contrast to the studies mentioned above, our study includes a two years follow-up of the subjects. Additionally, consistent to our observations is similar Swedish national study conducted by Ludvigsson et al., who showed in their one-year follow-up of children with newly diagnosed diabetes that subjects with higher BMI presented higher levels of C-peptide at diagnosis, whereas they lost more C-peptide during the first year after the onset of the disease than those with lower BMI (36). On the contrary, retrospective study of a small group of Korean children revealed that patients with lower BMI, significant symptoms of complications and a low C-peptide level at baseline, tended to show a rapid rate of decrease in C-peptide level (37).

Our observation showed that the third quartile BMI-SDS group experienced the smallest decrease in C-peptide level during the two-year follow-up, which may indicate that children with such body mass (mean BMI-SDS -0.72 ± 0.29) preserve β-cell function the most over the progression of type 1 diabetes. It is important to note that the first quartile BMI-SDS group showed almost the same decrease in C-peptide levels as the SDS-BMI >1 group which means that the leanest children do not preserve the function of the pancreatic islets in the best manner.

Furthermore, we found that children with BMI-SDS>1 were close to achieving criteria for PR after one year of implementing intensive insulin therapy, whereas two years after diagnosis their insulin requirements significantly increased, which may indicate that high BMI is not a factor that can contribute to prolonged PR period. In our relative risk analysis children with SDS-BMI above 1 occurred unfortunately to maintain increased body mass during the two years observation. This situation opens new therapeutic strategies – to emphasize the great need of normalization of body mass in those T1DM recognized with overweight. The negative effect of BMI on the preservation of pancreatic β-cell function in children with type 1 diabetes may be potentially explained by Wilkin’s accelerator hypothesis according to which the differences between type 1 diabetes and type 2 are blurred both clinically and etiologically (38). Therefore, a new term, double diabetes, has emerged to characterize people with type 1 diabetes who present clinical signs of type 2 diabetes like obesity and insulin resistance (6). Traditionally, type 1 diabetes sufferers are characterized as low or normal weight, with a little likelihood of metabolic syndrome or insulin resistance development, which are common features of type 2 diabetes (39). The accelerator hypothesis implies that the worsening of glucose control is the result of insulin resistance - the factor that accelerate the rate of β-cell loss through apoptosis (38). This phenomenon occurs through glucotoxicity and lipotoxicity and is responsible for what is conventionally known as type 2 diabetes (40). Importantly, it has been previously shown that obese, insulin-resistant patients with type 1 diabetes are likely to present with normal or elevated C-peptide levels at the time of diabetes diagnosis which is in line with our findings (41). Because of the fact that C-peptide is produced in equimolar amounts to endogenous insulin, its elevated levels are characteristic of insulin resistance and the metabolic syndrome phenotype (42), which are associated with hyperinsulinemia (43). Therefore, greater insulin resistance may account for higher levels of C-peptide observed in individuals with elevated BMI. In this situation, patients with higher BMI may experience symptoms that suggest insulin deficiency, despite having elevated insulin levels.

Interestingly, some studies show a positive correlation between BMI and levels of inflammatory cytokines (20, 26, 44). This is in line with our findings that subjects with SDS-BMI>1 present higher level of inflammatory interleukins than their peers with SDS-BMI <1. It was previously demonstrated that obese and overweight children with type 1 diabetes have low serum levels of IL-10, an interleukin that inhibits inflammatory responses in parallel with high levels of IL-17, a pro-inflammatory cytokine (44). Noteworthy, in our recent study we discovered that type 1 diabetic children with high plasma IL-17 levels present significantly increased SDS-BMI values (33). It is important fact that IL-17 augments inflammatory responses, plays a role in development of insulin resistance and intensifies autoimmune β-cell destruction (25). Another study found a positive correlation of TNF-α and IL-12, pro-inflammatory cytokines with BMI and obesity in patients with type 1 diabetes (26). In turn, Redondo et al. showed that obesity is associated with higher levels of TNF-α and lower levels of anti-inflammatory adipokines like omentin in children with newly diagnosed autoimmune type 1 diabetes (20). It has been demonstrated that TNF-α leads to insulin resistance and contributes to β-cell destruction (27). It has been also shown that obese individuals experience an increase in pro-inflammatory IL-1α and IL-1β, which consequently lead to insulin resistance (28). Interestingly, recent studies have shown that T1DM sufferers with worse glucose control or in the early stage of the disease demonstrate significant increase in IL-1β - cytokine which shows direct pathogenic impact on pancreatic islets. What is more, younger patients with T1DM present higher IL-1β levels which may be associated with cytokine storm during the first years of the disease (29). Unfortunately, we have not conducted any research on the origin of the cytokines we studied. In fact, they may originate from adipose tissue (45) but also from other cell populations, such as smooth muscle cells, endothelial cells or cardiomyocytes (46).

On the other hand, the correlation we have observed between BMI-SDS and GADA appears to confirm the destructive impact of obesity and its concominant low-grade inflammation on pancreatic beta cells *via* autoimmune mechanisms (47, 48). Our finding is in accordance with a recent study by Chylińska-Frątczak et al., which showed a 3.5-fold higher prevalence of at least one islet-specific autoantibody among obese children and adolescents compared to the general population (49).

The presence of a higher residual beta cell mass in individuals with type 1 diabetes could provide an opportunity to propose therapeutic interventions targeting the immune system in these patients. In development or under testing are several therapies aimed at modifying the immune system’s response to preserve beta cell function. Teplizumab, a CD3-directed monoclonal antibody was approved in November 2022 in the USA as a first drug indicated to delay the onset of stage 3 T1D in adults and pediatric patients aged 8 years and older with stage 2 T1D (50). Recent studies show that teplizumab treatment delays progression to T1D and improves beta cell function in high-risk individuals. The clinical response is associated with an increased frequency of KLRG1^+^TIGIT^+^CD8^+^ memory T cells, which show reduced IFNγ and TNFα secretion, which may indicate their functional exhaustion (51). Likewise, abatacept (CTLA4-Ig) have shown promising results in slowing the rate of reduction in beta cell function in individuals with recently diagnosed type 1 diabetes by blocking T-cell co-stimulation (52). It was also shown that golimumab, a monoclonal antibody against TNFα can have beneficial effects on endogenous insulin production and exogenous insulin requirements in children and young adults with newly diagnosed type 1 diabetes (53).

It should be added that the higher, unfavorable inflammatory milieu observed in patients with higher BMI may also reduce the efficiency of therapeutic interventions. It is proven that inflammation contributes to the development of insulin resistance (54), thus increasing the requirement for exogenous insulin. It has previously been shown that reducing BMI through physical activity reduces insulin resistance and increases the effectiveness of type 1 diabetes therapy (17).

Nevertheless, our study has some limitations. The study was performed in a single center, thus our group of patients was not large and diversified enough. Moreover, only 22 children were classified into the BMI-SDS >1 category, since the BMI-SDS <1 consisted of 121, the results might have been affected by statistical errors. We believe that also the correlation of cytokines and C-peptide levels do not show statistical significance because of the small study group. Therefore, a wider range of further research and epidemiologic studies with a greater number of varied patients are required. Additionally, the influence of factors that may affect C-peptide levels, such as history of infections and puberty (34, 55) was not considered. Low grade inflammatory state may have influenced the results and the decrease in C-peptide levels after 2 years of observation, but unfortunately we did not test hsCRP at the beginning, whereas CRP levels were within normal range. Unfortunately, we did not assess insulin resistance and cannot preclude the importance of individual insulin resistance or genetic predisposition for the C-peptide preservation at diagnosis. However, the mean and median age of the entire study group and individual groups were similar and prepubertal, hence the state of insulin resistance of these children could have been similar.

## 7 Conclusion

Higher BMI at type 1 diabetes onset in children, connected initially with preserved C-peptide, does not protect from rapid decrease in β-cell function in these patients. Significant decrease in residual β-cell function within two years from diagnosis in a group of children with the highest BMI may be the result of negative impact of elevated levels of inflammatory cytokines on pancreatic β-cells in the course of obesity. In terms of C-peptide preservation, it is most beneficial for type 1 diabetes sufferers to keep their BMI-SDS similar to individuals in the third quartile group of our follow-up, and it should be noted that neither the highest BMI-SDS nor the lowest BMI-SDS have a beneficial effect on preserving residual beta-cell function. Moreover, higher BMI is not associated with a greater likelihood of prolonged PR period. Since it has been proven that residual β-cell function has an advantageous result on metabolic control several years after disease onset this issue needs further investigation and prospective studies. Therapeutic strategies to maintain body mass within third centile of BMI-SDS ranges should be developed in order to prolong C-peptide, hence endogenous insulin secretion in new onset type 1 diabetes in children.

## Data availability statement

The raw data supporting the conclusions of this article will be made available by the authors, without undue reservation.

## Ethics statement

The studies involving human participants were reviewed and approved by Ethical Committee at the Medical University of Bialystok. Written informed consent to participate in this study was provided by the participants’ legal guardian/next of kin.

## Author contributions

Conceptualization, EK, SC and BG-O. Data curation, EK, SC, MJ-S and BG-O. Methodology, EK, SC, MJ-S, AP, AS and KG. Formal analysis, EK, SC, AS and KG. Investigation, EK, SC and MJ-S. Resources, EK, SC, MJ-S, AP and BG-O. Writing – original draft, EK and SC. Writing – review & editing, KG, MM, AB and BG-O. Visualization, BG-O. Validation, MM, AB and BG-O. Project administration, BG-O. Supervision, MM, AB and BG-O. Funding acquisition, BG-O. All authors contributed to the article and approved the submitted version.
